# Mesoporous Silica Particles Functionalized with Newly Extracted Fish Oil (Omeg@Silica) Reducing IL-8 Counteract Cell Migration in NSCLC Cell Lines

**DOI:** 10.3390/pharmaceutics14102079

**Published:** 2022-09-29

**Authors:** Claudia D’Anna, Caterina Di Sano, Serena Di Vincenzo, Simona Taverna, Giuseppe Cammarata, Antonino Scurria, Mario Pagliaro, Rosaria Ciriminna, Elisabetta Pace

**Affiliations:** 1 Istituto di Farmacologia Traslazionale, CNR, Via U. La Malfa 153, 90146 Palermo, Italy; 2 Istituto per lo Studio dei Materiali Nanostrutturati, CNR, Via U. La Malfa 153, 90146 Palermo, Italy

**Keywords:** omega-3, lung cancer cells, cancer cell migration, inflammation, fish oil, PUFA

## Abstract

Lung cancer is one of the leading forms of cancer in developed countries. Interleukin-8 (IL-8), a pro-inflammatory cytokine, exerts relevant effects in cancer growth and progression, including angiogenesis and metastasis in lung cancer. Mesoporous silica particles, functionalized with newly extracted fish oil (Omeg@Silica), are more effective than the fish oil alone in anti-proliferative and pro-apoptotic effects in non-small cell lung cancer (NSCLC) cell lines. The mechanisms that explain this efficacy are not yet understood. The aim of the present study is therefore to decipher the anti-cancer effects of a formulation of Omeg@Silica in aqueous ethanol (FOS) in adenocarcinoma (A549) and muco-epidermoid (NCI-H292) lung cancer cells, evaluating cell migration, as well as IL-8, NF-κB, and miRNA-21 expression. Results show that in both cell lines, FOS was more efficient than oil alone, in decreasing cell migration and IL-8 gene expression. FOS reduced IL-8 protein release in both cell lines, but this effect was only stronger than the oil alone in A549. In A549, FOS was able to reduce miRNA-21 and transcription factor NF-κB nuclear expression. Taken together, these data support the potential use of the Omeg@Silica as an add-on therapy for NSCLC. Dedicated studies which prove clinical efficacy are needed.

## 1. Introduction

Lung cancer is one of the most common diagnosed and harmful forms of cancer, above all in developed countries [1]. Amounting to approximately 15% of all lung cancers, small-cell lung cancer (SCLC) is characterized by poor clinical progress in therapy, also due to high proliferative rates [2]. Representing 85% of lung tumours, non-small-cell lung cancer (NSCLC) is conventionally classified on the basis of histological type, as well as squamous cell carcinoma (LUSC), large cell carcinoma, and adenocarcinoma (LUAD). LUAD represents the main diagnosed histological subtype, characterized by a poor response to therapies and reduced long-term survivals [1]. NSCLCs indeed arise from different cells and via different biological and molecular pathways [3].

In this context, we recently reported that mesoporous silica particles, functionalized with newly extracted fish oil (Omeg@Silica), significantly inhibit (in vitro) NSCLC cell growth [4]. In closer detail, when tested on lung adenocarcinoma A549 and Colo699 cells, a hydroalcoholic formulation of “Omeg@Silica” submicron particles in aqueous ethanol (FOS) was found to be significantly more active than the fish oil alone in terms of cell apoptosis, long-term proliferation, and mitochondrial reactive oxygen species (ROS) production [4].

The new Omeg@Silica material is made of a whole fish oil (AnchoisOil) sustainably sourced from anchovy fillet leftovers with citrus limonene [5], encapsulated within the inner mesoporosity of periodic mesoporous Mobil Composition of Matter No. 41 (MCM-41) silica submicron particles [6]. Obtained from citrus peel, *d*-limonene is a biobased solvent endowed with several health-benefitting properties [7].

The molecular mechanisms that explain this remarkable efficacy of said Omeg@Silica submicron particles in aqueous ethanol, not yet understood, need to be urgently identified in light of the numerous promising characteristics of this new material. Its components (mesoporous silica, whole fish oil rich in polyunsaturated fatty acids, vitamin D3, and residual limonene) are indeed highly biocompatible and actually used in several commercial nutraceutical and pharmaceutical products.

The main polyunsaturated fatty acids (PUFAs) in fish oil are docosahexaenoic acid (DHA, C22:6*n*-3) and eicosapentaenoic acid (EPA, C20:5*n*-3). In natural fish oil (i.e., not chemically refined, as is the case with most omega-3 food supplements), said PUFAs are present in triglyceride form. By significantly inhibiting tumour growth, suppressing cancer cell viability, and inducing apoptosis in various cancer cells, these essential lipids exert a therapeutic role against certain types of cancer [8], including lung cancer [9,10].

Tumour progression and formation result from a complicated network, in which different cell populations and signals take part. Interleukin-8 (IL-8, an inflammatory cytokine) is one of the key promoters of tumour progression given its ability to promote the angiogenic response of endothelial cells; recruit immunosuppressive cells (such as neutrophils); and induce the proliferation, survival, and migration of cancer cells [11]. Clinical trials in lung cancer patients have shown that patients with a high blood IL-8 concentration have worse outcomes and lower overall survival rate [12].

IL-8 production is regulated by different signalling pathways. Nuclear factor-kappa B (NF-κB) and mitogen-activated protein kinase (MAPK) are the main triggers associated with lung inflammation [13]. NF-κB, for instance, is one of main transcription factors involved in the inflammatory response of lung cells to cigarette smoke [14]. Regulating the survival, activation, and differentiation of inflammatory T cells, NF-κB controls, and is controlled (in a positive feedback mechanism), by the expression of microRNAs in a cycle involved in the regulation of pro-inflammatory response [15].

Thus, in this study, we explored the anti-cancer properties of Omeg@Silica microparticles using NSCLC cells lines which originate from different geographical districts of the lung, in particular adenocarcinoma (A549) and mucoepidermoid (NCI-H292) lung cancer cell lines, by evaluating cell migration, as well as the expression/release of IL-8, NF-κB, and miRNA-21.

## 2. Materials and Methods

### 2.1. Extraction of AnchoisOil

Fish oil was obtained from anchovy fillet leftovers gently given by an anchovy fillet company (Agostino Recca Conserve Alimentari, Sciacca, Italy), according to a circular economy process introduced in 2019, using biobased *d*-limonene as an extraction solvent [5]. An oil, in a deep orange colour, was obtained (AnchoisOil) containing a high quantity (81.5 μg/kg) of vitamin D in the form of bioactive isomer vitamin D_3_ (cholecalciferol).

### 2.2. Preparation of Omeg@Silica

The Omeg@Silica particles used in this study were MCM-41 silica particles loaded with 50 wt% anchovy fish oil. Their synthesis via the microencapsulation of AnchoisOil, following the dropwise addition of oil to the mesoporous silica, was carried out, as previously described [6]. The aqueous formulations of diverse concentrations used in this study were obtained by appropriate dilution of mother suspensions of Omeg@Silica, AnchoisOil, and MCM-41 in 10 *v*/*v*% ethanol–PBS Dulbecco’s phosphate-buffered saline (Euroclone, Pero, Italy). In particular, the Omeg@Silica mother suspension was prepared by adding 10.1 mg of material to 1 mL of ethanol and 9 mL of PBS. This formulation of Omeg@Silica microparticles in aqueous ethanol is named FOS. The AnchoisOil and the MCM-41 mother suspensions had a concentration of the single components equivalent to that of the Omeg@Silica mother suspension. Then, 5 mg of AnchoisOil was suspended in 10 mL of 10 *v*/*v*% ethanol–PBS and 5.1 mg of MCM-41 was suspended in 10 mL of 10 *v*/*v*% ethanol–PBS.

### 2.3. Cell Culture and Treatment

A549 and NCI-H292 cells were purchased from Interlab Cell Line Collection (Genoa, Italy). The cells were kept in RPMI with 10% heat-inactivated fetal calf serum, 100 U/mL of penicillin, 100 μg/mL of streptomycin, 1% MEM non-essential amino acids, 2 mM of l-glutamine, and 250 μg/mL of gentamicin, at 37 °C in a humidified atmosphere containing 5% CO_2_. In the sub-confluent cell monolayer, the serum concentration in the medium was reduced to 1%, and then was treated with oil. Following this, FOS (10 μg/mL) was performed.

In order to evaluate possible effects due exclusively to microparticles, we treated cells with silica alone. The concentration of FOS (10 μg/mL) used in the present work was identified in a previous work on A549 where cell viability, necrosis, and cell apoptosis were assessed [4]. Cell viability data in H292 are shown in Figure S1. The cells were harvested immediately after 3 h of stimulation to investigate the wound-healing assay (as cell motility test) after 24 h to assess NF-κB expression, IL-8 release, as well as IL-8 and miRNA-21 gene expression.

### 2.4. Wound-Healing Assay

A549 and H292 cells were seeded in a 6-well plate and were cultured to confluence. Three circular wounds were prepared in each well using a 200-µL pipette tip as previously described [16]. After washing with PBS to eliminate debris, cells were allowed to recover for one hour and then stimulated with oil, silica, FOS (10 μg/mL), and anti-IL-8 (1 μg/mL) (R&D Systems, Minneapolis, MN, USA), as already described. Images were captured using a digital camera connected to an inverted phase-contrast optical microscope 0, 24, and 48 h after wound creation. The wound area was measured using the ImageJ program in order to assess the remaining wound sizes and wound closure rates.

### 2.5. RNA Extraction

The total RNA was extracted by A549 and H292 cell lines, treated or not with oil, FOS, and silica, using the RNAspin mini kit (GE Healthcare Science, Uppsala, Sweden), according to the manufacturer’s protocol. The RNA concentration was assessed using the SpectroStar Nano reader (BMG Labtech, Ortenberg, Ortenberg, Germany). For this study, we only used the RNA with a ratio of A260/280 from 1.9 to 2.

### 2.6. TaqMan RT-qPCR for IL-8 and miRNA-21-5p

From the total RNA, the cDNA was synthesized using the iScript cDNA synthesis kit (Biorad), according to the manufacturer’s protocol. IL-8 gene expression was evaluated with specific FAM-labelled probe and primers, as part of the TaqMan gene expression assay for IL-8 (Hs00174103m1 Applied Biosystems, Foster City, CA, USA) with qRT-PCR using a Step One Plus real-time PCR system (Applied Biosystems, Foster City, CA, USA). The gene expression was normalized to GAPDH with a Taq-Man gene expression assay for GAPDH (Hs03929097_g1, Applied Biosystems, Foster City, CA, USA) as a housekeeping gene. For the normalization of RT-qPCR data, the 2-ΔCT method was used.

For the reverse transcription of miRNA-21-5p, 1 µg of total RNA was incubated with specific primers using the TaqMan™ microRNA assay (Assay ID: 000397, Applied Biosystems, Foster City, CA, USA) and the TaqMan miRNA RT kit (Applied Biosystems, Foster City, CA, USA), according to the manufacturer’s protocol. miRNA-21-5p expression was evaluated using specific TaqMan microRNA assays (Assay ID: 000397, Applied Biosystems, Foster City, CA, USA) with qRT-PCR and the Step One Plus real-time PCR system (Applied Biosystems, Foster City, CA, USA). MiRNA-21-5p expression was normalized with RNU6 using the TaqMan™ microRNA assay (Assay ID: 001973, Applied Biosystems, Foster City, CA, USA), as endogenous control. For the normalization of RT-qPCR data, the 2-ΔCT method was used.

The results were expressed as a percentage of area reduction at time point 24 h (T24h) and 48 h (T48h), compared to time point 0 h (T0h).

### 2.7. IL-8 Protein Release

The release of IL-8 was evaluated by the ELISA method with the enzyme-linked immunosorbent assays (DuoSet R&D Systems, Minneapolis, MN, USA), according to the manufacturer’s instructions. Cell supernatants of both line cells were harvested after 24 h of stimulation, centrifuged for 15 min at 15,000× *g*, and stored at −80 °C until the assay was used. These experiments were performed in triplicate.

### 2.8. NF-κB Expression by Western Blot Analysis

Nuclear translocation of NF-κB was assessed by Western blot analysis. Three hours after treatment with oil, silica, and FOS, A549 and H292 pellets were collected, and cytoplasmic and nuclear proteins were extracted using a commercial kit (a nuclear and cytoplasmic extraction kit from Thermo Scientific Waltham, MA, USA), as previously described [17]. Protein concentrations were evaluated using the Bradford assay (Biorad, Hercules, CA, USA). Then, 30 μg of nuclear proteins was separated using 4–15% Mini-PROTEAN TGX precast protein gels, before then being transferred onto a nitrocellulose membrane (Hybond C). Western immunoblotting was assessed with a polyclonal rabbit antibody diluted 1:200 in milk, directed against p65/NF-κB (C-20: sc-372, Santa Cruz Biotechnology, Santa Cruz, CA, USA). Specific bands were highlighted using the enhanced ECL Western blotting analysis system (Amersham Pharmacia Biotech, now GE Healthcare Life Sciences). For the loading control, membranes were re-tested with a mouse anti-*β*-actin mAb (1/10,000; Sigma, St. Louis, MO, USA). These experiments were performed in triplicate.

### 2.9. Statistical Analysis

The determination of statistical significance for differentially expressed signals and for the scratch test assay was performed using an analysis of variance (ANOVA) approach corrected with Fisher’s test. A value of *p* < 0.05 was regarded as statistically significant.

## 3. Results

### 3.1. Effects of Fish Oil, Silica, and FOS on Cell Migration in A549 and NCI-H292 Cells

**Figure 1:**
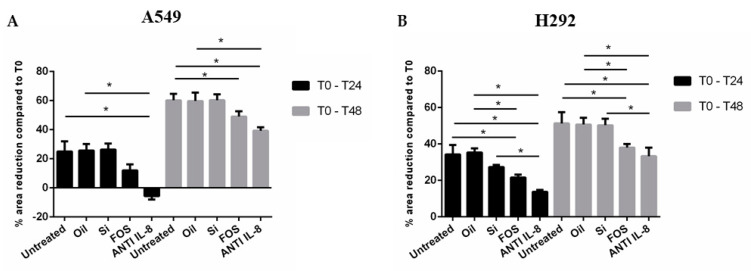
Effects of FOS in cell migration. A549 (**A**) and NCI-H292 (**B**) were stimulated with fish oil, silica, FOS (10 μg/mL), and anti-IL-8 for 24 and 48 h, and cell motility was assessed using the wound-healing test. We used anti-IL-8 (1 μg/mL) to further demonstrate the central role in the cell migration of this cytokine. The results were expressed as a percentage of area reduction at 24 h and 48 h compared to time point 0 h (n = 3) ± SD. The comparison between different experimental conditions was evaluated by ANOVA corrected with Fisher’s test. * *p <* 0.05 was accepted as statistically significant.

Increased cancer cell motility is a feature of tumour aggressiveness [18]. To evaluate whether the stimulation with FOS reduced cell motility, we used the wound-healing test. Cells were stimulated with fish oil, silica, and FOS (10 μg/mL) for 24 and 48 h. A blocking antibody of IL-8 activity was included to assess the specific role of IL-8 in cancer cell motility. A scratch was made in each well using a 200 μL pipette tip. As shown in Figure 1A,B, at 24 and 48 h in both cell lines, FOS and anti-IL-8 were able to significantly reduce cell motility and FOS was more effective than fish oil alone. No significant difference was detected between untreated cells and oil- and silica-treated cells.

### 3.2. Effects of Fish Oil, Silica, and FOS on IL-8 Gene Expression and Release, in A549 and NCI-H292 Cells

**Figure 2:**
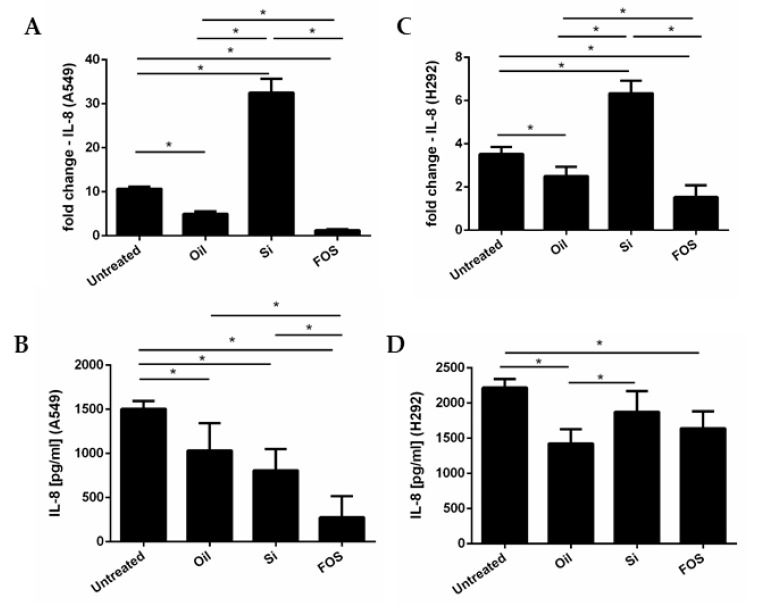
Effects of FOS on the expression of IL-8 mRNA and protein release. A549 and NCI-H292 were cultured with fish oil, silica, and FOS (10 μg/mL) for 24 h; total RNA was extracted; and supernatants were collected to assess IL-8 gene expression by real-time PCR and IL-8 protein release using ELISA, respectively. (**A**,**B**) report data on IL-8 gene expression and IL-8 protein release in A549 cells, respectively. (**C**,**D**) report data on IL-8 gene expression and IL-8 protein release in NCI-H292, respectively. Results are expressed as mean ± SD (n = 3). A comparison between different experimental conditions was evaluated by ANOVA corrected with Fisher’s test. * *p <* 0.05 was accepted as statistically significant.

A549 and NCI-H292 were cultured with fish oil, silica, and FOS (10 μg /mL). After 24 h, the total RNA was extracted and supernatants were collected. To assess IL-8 gene expression, real-time PCR was used. The IL-8 concentration in cell supernatants was evaluated with an ELISA kit. As shown in Figure 2A,B, in A549, FOS was able to significantly reduce IL-8 gene expression and protein release, more than fish oil alone. Regarding NCI-H292 cells (Figure 2C,D), FOS significantly decreased IL-8 gene expression more than fish oil alone, but significantly reduced IL-8 release with the same extent of fish oil. Although silica significantly induced IL-8 gene expression (Figure 2A–C), it did not significantly increase IL-8 release in both cell lines (Figure 2B–D).

### 3.3. Effects of Fish Oil, Silica, and FOS on NF-κB Expression in A549 and NCI-H292 Cells

**Figure 3:**
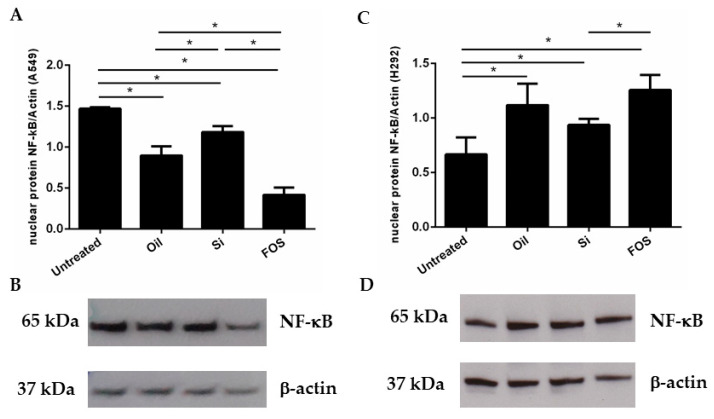
Effects of FOS in NF-κB expression. A549 and NCI-H292 were cultured with fish oil, silica, and FOS (10 μg/mL) for 24 h. The nuclear expression of NF-κB was assessed by Western blot analysis on isolated nuclear protein fractions. (**A**–**C**) Signals corresponding to NF-κB on the various Western blots were semi-quantified by densitometric scanning, normalized, and expressed after correction with the density of the band obtained for *β*-actin. Bars represent the fold change, expressed as the mean ± SE. Graph A shows data on A549, while Graph C shows data on H292. The comparison between different experimental conditions was evaluated by ANOVA corrected with Fisher’s test. * *p* < 0.05 was accepted as statistically significant. (**B**–**D**) Representative Western blots were shown for A549 (**B**) and for H292 (**D**). Lane 1: untreated; lane 2: oil 10 μg/mL; lane 3: silica 10 μg/mL; lane 4: FOS 10 μg/mL).

Since NF-κB activation can control IL-8 expression [13], it was investigated whether FOS affected NF-κB activation by evaluating the nuclear expression of NF-κB via Western blot analysis in cells stimulated with oil, silica, and FOS (10 μg /mL). As shown in Figure 3A,B, FOS significantly decreased NF-κB expression in A549 only, in comparison to untreated cells and fish-oil-treated cells. Fish oil and silica treatments did not induce significant differences. In NCI-H292, no significant difference was observed (Figure 3C,D).

### 3.4. Effects of Fish Oil, Silica, and FOS on miRNA-21 Expression in A549 and NCI-H292 Cells

**Figure 4:**
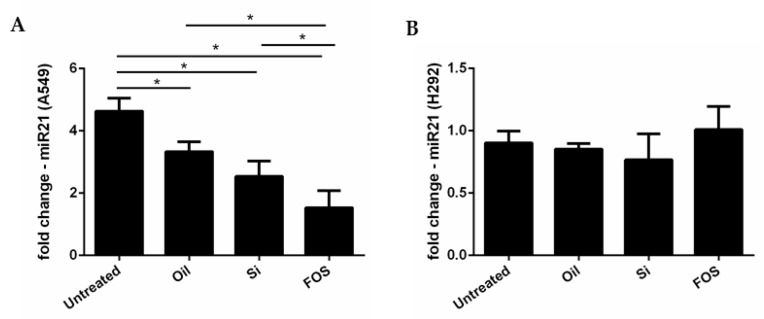
Effects of FOS in miRNA-21 gene expression. A549 (**A**) and H292 (**B**) were treated with oil, silica, and FOS for 24 h, and miRNA-21 expression was assessed by real-time PCR. Results are expressed as mean ± SD (n = 3). The comparison between different experimental conditions was evaluated by ANOVA corrected with Fisher’s test. * *p* < 0.05 was accepted as statistically significant.

In A549, IL-8 gene expression and release increased using miR-21 up-regulated expression [15]. We treated A549 and H292 with oil, silica, and FOS (10 μg/mL) for 24 h, and miRNA-21 expression was assessed. In A549, we found that FOS, silica, and oil were able to significantly reduce miRNA-21 expression, and FOS was more effective than fish oil alone (Figure 4A). In H292, miRNA-21 expression was not modified (Figure 4B).

## 4. Discussion

Lung cancer is a very aggressive cancer. About 80% of lung cancers are non-small cell lung cancer (NSCLC) which mainly consists of two subtypes: lung adenocarcinoma (LUAD) and lung squamous cell carcinoma (LUSC). They arise from different cells and differ in prognosis, signalling pathways, and gene expression. Moreover, LUAD provides about 40% of all diagnosed lung cancers [19]. NSCLC patients generally have a poor prognosis, with a 5-year survival rate of less than 18% and approximately 0–10% for patients in advanced stages [20]. Despite continuing advances in personalized therapy and immunotherapy, only some subgroups of patients benefit from these treatments, although most of them tend to develop drug resistance [18,21], and new lung cancer therapies are needed.

Submicron mesoporous silica particles, functionalized with whole fish oil extracted with limonene only (Omeg@Silica) and further dispersed in aqueous ethanol (FOS), have recently been demonstrated to exert in vitro anti-tumour effects on NSCLC cell lines, increasing mitochondrial ROS production and cell apoptosis, and reducing cell growth ability.

Herein, we further investigated the anti-cancer effects of FOS in two LUAD cancer cell lines which originate from different districts: adenocarcinoma (A549) and mucoepidermoid (NCI-H292) cell lines. This involves evaluating IL-8, NF-κB, and miRNA-21 expression, as well as molecules associated with cell migration (a process with a relevant role in cancer progression).

In lung cancer, it was demonstrated that ω-3 PUFAs and their metabolites regulate pathways controlling the progression of lung cancer. Trombetta et al. showed that in A549, ω-6 and ω-3 PUFAs have anti-proliferative effects, affecting the DNA-binding activity of AP-1 and inducing PPAR [22]. In addition, on A549, Zajdel et al. demonstrated that both EPA and DHA suppress lung cancer cell growth and improve cell death [23].

Further, 17-oxo-DHA has antitumor effects in NSCLC cell lines and, in combination with gemcitabine, leads to stronger anti-cancer effects compared to gemcitabine alone [24].

The connection between chronic inflammation and tumour growth is well established [25]. It represents one of the most crucial epigenetic and environmental causes, promoting tumour progression [26]. Therefore, removing (or reducing) inflammation may be essential for cancer prevention and therapy.

Upon dietary consumption, long-chain polyunsaturated fatty acids (PUFAs), especially ω-3 docosahexaenoic acid (DHA) and eicosapentaenoic acid (EPA), commonly found in fish oils, have helpful effects against inflammation. Clinical studies on the dietary intake of PUFAs and in vitro studies showed that DHA can significantly decrease the expression of inflammatory cytokines and the expression of genes involved in inflammatory pathways such as NF-κB [27]. A pivotal step to establish the progression of a tumour is the obtainment of aggressive characteristics by carcinoma cells. Increased inflammation can support the achievement of aggressive behaviour, leading to metastasis, the prevalent cause of death from cancer. During the process of metastasis, tumour cells leave the primary site and spread throughout the body, forming secondary sites. Several different mechanisms in the tumour microenvironment influence the regulation of cancer cell migration and invasion, including the presence of hypoxia, chemo-attractants, and epithelial–mesenchymal transition (EMT) processes. EMT promotes the switch from a non-motile epithelial to a motile mesenchymal state, endowing cancer cells with multiple malignant features, such as the increased invasiveness and resistance to senescence, apoptosis, and treatment. In the tumour microenvironment, IL-8, supporting all the above-cited events, appears markedly important and has been presented as one of the prominent promoters of tumour progression.

Taking these findings into account, we tested whether FOS exerted anti-cancer effects, decreasing cell migration via the scratch assay. For the first time, we found that, both in A549 and H292, FOS decreases cell migration, more than in fish oil alone, and the FOS effects were similar to the effect exerted by anti-IL-8. These findings prompted us to test the effect of FOS, oil, and silica treatments on IL-8 expression and release in both cell lines. As expected, FOS was able to reduce IL-8 gene expression and protein release both in A549 and NCI-H292. Only in A549, FOS was more efficient in reducing both IL-8 gene expression and protein release than fish oil alone. In A549 cells, ω-3 fatty acids significantly decreased IL-8 [28]. Here, for the first time, we show that the use of whole fish oil encapsulated in silica particles (FOS) is more efficient than fish oil alone in reducing IL-8 expression in a cell-specific manner. Although silica alone increased IL-8 gene expression in both cell lines, this effect was not biologically relevant because it is not associated with the increased release of IL-8. In addition, the gene expression and release of IL-8 in FOS treated cells were reduced in comparison to silica-treated cells. We found that the mostly spherical Omeg@Silica particles with their inner mesoporosity filled with 50 wt% fish oil were 269 nm in size and had both a low (0.3) polydispersity index and a large negative (−37.6 mV) value of zeta potential [6]. Originating from an electrostatically stable dispersion once dispersed in water or in aqueous ethanol, the latter negative Z-potential beyond −30 mV is a crucially important characteristic to develop effective drug delivery applications [6]. The encapsulation of AnchoisOil fish oil in mesoporous silica particles dispersed in aqueous ethanol thus seems to change the mechanism of action of the new whole fish oil sustainably extracted from anchovy fillet leftovers with bio-solvent limonene [5], resulting in different anticancer activity mechanisms. We ascribe such a remarkable change in the activity of encapsulated PUFAs in triglyceride form to the much higher bioavailability of the silica-encapsulated lipid molecules when compared to the free AnchoisOil.

It is well known that the hydrophilic–lipophilic balance (HLB) of cancer cell membranes is altered, with an increase in cell permeability and enhanced hydrophilic character. Hence, for example, the uptake of hydrophobic curcumin microencapsulated by A549 cells is highest for submicron pluronic-stabilized gelatin particles with the lowest hydrophobicity [29]. We make the hypothesis that the cellular uptake of both hydrophobic PUFA molecules in triglyceride form (and vitamin D_3_) [30] of AnchoisOil, encapsulated and stabilized in the inner porosity of the highly hydrophilic mesoporous silica via endocytosis and direct translocation [31], is significantly enhanced when compared to the free hydrophobic molecules in the hydrophilic cellular medium. Moreover, in agreement with previous knowledge on sol–gel-entrapped materials [32], we assume that the PUFA triacylglycerol and vitamin D_3_ molecules entrapped in the silica pores are protected from chemical oxidative degradation due to the air’s oxygen and natural light.

The overexpression of IL-8 is present in different types of cancer, including multiple myeloma, hepatocarcinoma, chronic lymphocytic leukemia [33,34,35,36,37] melanoma, colon carcinoma, pancreatic cell lines [33,34,35,36,37,38], and lung cancer [39]. In the same type of lung cancer, IL-8 concentrations is linked to tumour progression, survival, timing, and relapse [39]. In NSCLC, IL-8 promotes the angiogenic response of endothelial and epithelial cells [40,41].

NF-κB is the main regulator of pro-inflammatory gene expression, particularly IL-8 expression [42]. In the related pathway, NF-κB is a homo- or heterodimer usually consisting of the p65/p50 dimer. In resting cells, it is bound to IκB, remaining inactivated in the cytosol. Following stimulation, IκB is phosphorylated, ubiquitinated, and degraded, allowing p65/p50 to translocate to the nucleus and bind to κB sites in promoters of target genes [43].

Hence, we assessed the nuclear expression of NF-κB in both cell lines. Only in A549 cell line, FOS was more efficient than fish oil alone to induce a significantly impairment in NF-κB expression. In the H292 cell line, under the same conditions, NF-κB expression was unaltered. These findings suggest that in H292, the effect of FOS on IL-8 gene expression and release can be due to the modulation of different transcription factors and kinase cascades, such as C/EBP-1, CREB, or AP-1, which bind to the IL-8 promoter [44].

Remarkably, it has already been demonstrated that in different uroepithelial cells, uropathogenic *E. coli* induces a differential IL-8 gene regulation pathway [45], with cell-type-specific mechanisms [46]. Similarly, the use of poly-(propyleneimine) (PPI) glycodendrimers as potential immunomodulatory induces IL-8 expression in a different way in HL-60 and THP-1 cells (via AP-1 binding and mRNA stabilization, respectively) [47]. Furthermore, it is noteworthy that a great variance of cellular control networks, such as biological determinants or tumour progression mechanisms, can induce transformation in adenocarcinomas versus squamous cell lung cancers [48].

In this regard, in the same subgroup of lung cancer (NSCLC), Sorrenti and co-workers have shown that tin mesoporphyrin (SnMP) has different anti-cancer effects in A549 and NCI-H292, likely due to gene level regulation by miRNA [49]. As stated above, different biological pathways have been highlighted, such as the overexpression of COXO2 in mucoepidermoid cells (NCI-H292) with respect to adenocarcinoma cells (A549) [2] and a higher expression of HO-1 in A549 cells in comparison with H292 cells.

These differences can be explained by the fact that these cancer cells have a different origin: H292 originates from the minor salivary glands lining the tracheobronchial tree, while A549 is derived from bronchioalveolar stem cells or from alveolar type 2 (AT2) cells. A significant relationship between microRNAs and IL-8 exists: at the post-transcriptional level, IL-8 is controlled by the miRNA network [50]. In addition, in NSCLC NF-κB upregulates the expression of miRNA-21, through the binding of NF-κB to the miRNA-21 promoter region [51].

MiRNA-21, an onco-miR, represents a fundamental link between cancer and inflammation, as its increased expression has been found in different cancers. In lung cancer, specifically in NSCLC, several studies report on the upregulated levels of miRNA-21 in diagnosed patients. The affected expression of miRNA-21 disturbs different pathways, including cell proliferation and angiogenesis, as well as chemo- and radio-resistance [52].

The use of miRNA-21 antisense sequences as therapeutic modulators shows encouraging results in the management of NSCLC [52]. Thus, we assessed the expression of miRNA-21 in both cell lines. FOS, only in A549, reduces miRNA-21, and this reduction is more effective than fish oil alone.

## 5. Conclusions

In conclusion, after studying in vitro the effect of whole fish oil encapsulated in mesoporous silica formulated in aqueous ethanol on adenocarcinoma (A549) and mucoepidermoid (NCI-H292) lung cancer cell lines, we discovered that a formulation comprised of submicron mesoporous silica particles functionalized with whole fish oil extracted with limonene only (Omeg@Silica) dispersed in aqueous ethanol (FOS) reduces IL-8 release, affecting cell migration and cancer progression in lung cancer cells. In adenocarcinoma cells, FOS inhibits cell migration through the inhibition of NF-κB and miRNA-21 in a cell-type-specific manner. Offering the first mechanistic insight into deciphering FOS anti-cancer property, these findings delineate a novel pharmacological and therapeutic strategy for lung cancer patients based on whole fish oil derived from the most abundant fish species (the anchovy) across the world’s seas.

## Data Availability

Not applicable.

## Supplementary Materials

The following supporting information can be downloaded at: https://www.mdpi.com/article/10.3390/pharmaceutics14102079/s1. Figure S1: Cell viability in H292.
