# Supplementary material for: Mesoporous Silica Particles Functionalized with Newly Extracted Fish Oil (Omeg@Silica) Reducing IL-8 Counteract Cell Migration in NSCLC Cell Lines
Source: Pharmaceutics. 2022 Sep 29;14(10):2079. doi: 10.3390/pharmaceutics14102079 (PMC9609990; doi:10.3390/pharmaceutics14102079)
Supplement: Supplementary file 1 [file pharmaceutics-14-02079-s001.zip › pharmaceutics-1893791-supplementary.pdf]

pharmaceutics

MDPI

# Supplementary Materials: Mesoporous Silica Particles Functionalized with Newly Extracted Fish Oil (Omeg@Silica) Reducing IL-8 to Counteract Cell Migration in NSCLC Cell Lines

**Claudia D'Anna, Caterina Di Sano, Serena Di Vincenzo, Simona Taverna, Giuseppe Cammarata, Antonino Scurria, Mario Pagliaro, Rosaria Ciriminna and Elisabetta Pace**

**Cell viability assay**

To determine the cell viability of H292 cell cultures, we used a colorimetric method (CellTiter 96® Aqueous One Solution Cell Proliferation Assay, PROMEGA, Madison WI USA). This approach can determine viable cells in proliferation, chemosensitivity, or cytotoxicity assays. Cells were plated in 96-well plates and were treated for 24 h in duplicate with AnchoisOil dispersed in ethanol (fish oil) (10 µg /ml), silica sub-micron particles (10 µg /ml), and AnchoisOil encapsulated in silica (FOS) (10 µg /ml), and the test was performed according to manufacturer's protocol. The microplate reader WallacVictor2 1420 Multilabel Counter (Perkin Elmer) was used to read the absorbance at 490 nm. The results were calculated as the percentage of absorbance with respect to that of the control (untreated cells). For the A549 cells, the same test was performed in our previous work [4].

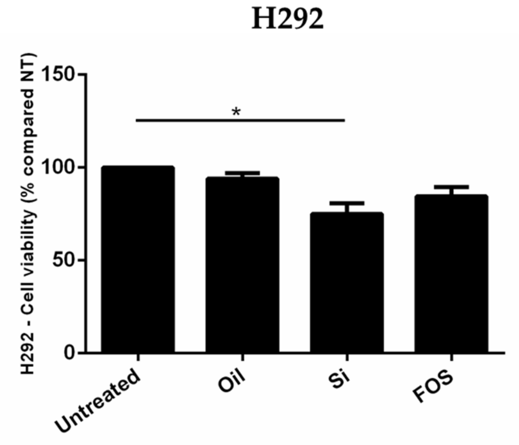


**Figure S1.** Cell viability in H292. We treated H292 with oil, silica, and FOS, and cell viability was assessed by MTS. Only silica was able to significantly reduce cell viability. Results are expressed as mean ± SD (n = 2). A comparison between different experimental conditions was evaluated using a paired test. * $p < 0.05$ was accepted as statistically significant.

4 Di Sano, C.; D'Anna, C.; Scurria, A.; Lino, C.; Pagliaro, M.; Ciriminna, R.; Pace, E. Mesoporous silica particles functionalized with newly extracted fish oil (Omeg@Silica) inhibit lung cancer cell growth. *Nanomedicine* **2021**, *16*, 2061–2074. https://doi.org/10.2217/nnm-2021-0202.
